# Role of pulsatile growth hormone (GH) secretion in the regulation of lipolysis in fasting humans

**DOI:** 10.1186/s40842-022-00137-y

**Published:** 2022-02-01

**Authors:** N. Goldenberg, J. F. Horowitz, A. Gorgey, A. Sakharova, A. L. Barkan

**Affiliations:** 1 Department of Medicine, University of Michigan, Ann Arbor, USA; 2 School of Kinesiology, University of Michigan, Ann Arbor, USA; 3 Departments of Medicine and Neurosurgery, Division of MEND, University of Michigan, 24 Frank Lloyd Wright Drive G-1500, Ann Arbor, MI 48106 USA

**Keywords:** Somatotropin, Pulsatility, Starvation, Ketosis

## Abstract

**Background:**

The increase in growth hormone (GH) secretion during a prolonged fast stimulates lipolytic rate, thereby augmenting the mobilization of endogenous energy at a time when fuel availability is very low.

**Study aim:**

To identify the specific component of GH secretory pattern responsible for the stimulation of lipolytic rate during fasting in humans.

**Study protocol:**

We measured lipolytic rate (using stable isotope dilution technique) after an overnight fast in 15 young, healthy, non-obese subjects (11 men and 4 women), and again on four separate occasions after a 59 h fast. These four prolonged fasting trials differed only by the contents of an infusion solution provided throughout the 59 h fasting period. Subjects were infused either with normal saline (“Control”; *n* = 15) or with graded doses of a GH Releasing Hormone Receptor Antagonist (GHRHa):10 μg/kg/h (“High”; *n* = 15), 1 μg /kg/h (“Medium”; *n* = 8), or 0.5 μg /kg/h (“Low”; *n* = 6).

**Results:**

As expected, the 59 h fast completely suppressed plasma insulin levels and markedly increased endogenous GH concentrations (12 h vs 59 h Fast; *p* = 0.0044). Administration of GHRHa induced dose-dependent reduction in GH concentrations in response to the 59 h fast (*p* < 0.05). We found a strong correlation between the rate of lipolysis and GH mean peak amplitude (R = 0.471; *p* = 0.0019), and total GH pulse area under the curve (AUC) (R = 0.49; *p* = 0.0015), but not the GH peak frequency (R = 0.044; *p* = 0.8) or interpulse GH concentrations (R = 0.25; *p* = 0.115).

**Conclusion:**

During prolonged fasting (i.e., 2–3 days), when insulin secretion is abolished, the pulsatile component of GH secretion becomes a key metabolic regulator of the increase in lipolytic rate.

## Introduction

Growth hormone (GH) has major effects on metabolic processes in humans [1–5]. Traditionally, the role of GH in human metabolism was determined by infusing GH to patients with GH deficiency, mostly to patients with panhypopituitarism [1–5]. Those studies demonstrated that augmentation of lipolysis is the primary target of GH action in adults. Additionally, the development of insulin resistance and partial alleviation of the negative protein balance were secondary to enhanced fatty acid metabolism [2, 3]. However, GH infusions were not able to reproduce the physiological pulsatile pattern of GH presentation to the peripheral tissues. The use of a specific competitive GHRH receptor antagonist (GHRHa) [6] allowed to suppress daily GH output in a physiological dose-dependent fashion [7] and provided a novel tool to study the modulation of the metabolic parameters by endogenous GH in normal individuals.

Earlier, we have shown that GHRHa was capable of suppressing mean daily GH concentrations in healthy non-obese subjects to ~ 70% both in the fed and fasting state [8]. However, this did not alter the rate of lipolysis after a physiological overnight fast, but powerfully suppressed it after a total of 59 h fasting, when insulin levels were largely undetectable [8]. Thus, there is an interplay between GH and insulin in their respective functions as regulators of lipolysis: GH is lipolytic and insulin is antilpolytic hormone We have previously shown that the lipolytic effect of GH is expressed when insulin secretion is diminished, i.e. during fasting state [8]. This has major implication in the context of energy availability during starvation and/or other prolonged episodes of low caloric intake.

Our earlier findings demonstrated that in the absence of insulin, GH becomes the main metabolic hormone that is responsible for the required metabolic response to prolonged fasting, i.e. a shift from carbohydrates to fat as the main source of energy. Several studies have also shown that not only the total amount of GH secreted during the day, but also the pattern of GH presentation to peripheral tissues is important for its hormonal activity to be manifest [9]. In our earlier study involving obese subjects (the model of impoverished GH secretion) administration of physiological doses of GH in a continuous fashion selectively augmented both hepatic production of IGF-1 and muscle IGF-1 mRNA levels, whereas pulsatile administration of GH in the same daily dose preferentially augmented the rate of lipolysis [10].

To this end, we have employed a model of prolonged fasting in humans (59 h fast), during which insulin is maximally suppressed, while 24 h plasma GH concentration and GH pulsatility are naturally augmented [8]. Graded blockade of GHRH receptors by a specific competitive GHRHa and quantification of discrete parameters of GH pulsatility allowed us to pinpoint the relative influence of pulsatile vs. basal GH secretion as potential regulators of the rate of lipolysis in healthy humans.

## Methods

Subjects: Study population comprised 15 subjects: 11 healthy men and 4 healthy women, 18–46 years of age, mean (SE) age 26 ± 2 years, weight 77.3 kg ± 3.0 kg, BMI 20–24 kg/m^2^. Data from 6 subjects previously studied on an identical protocol were re-analyzed to assess their GH pulsatile parameters and were included in the current study [8] Exclusion criteria for participation in this study included: evidence of liver, renal, endocrine or cardiovascular disease, hyperlipidemia, hematocrit < 34%, medications known to alter GH secretion or action, lipid, glucose, and/or protein metabolism, pregnancy or breastfeeding. The protocol was approved by the IRB and the GCRC of University of Michigan and the written consent was obtained from all participants.

### Study protocol

This study consisted of a total of 5 separate experimental trials. During one trial, subjects were admitted for 24 h, during which they were provided standardized meals during the day of admission, and then we performed our battery of measurements (See details below) after an overnight fast (“12 h fast” trial). The remaining 4 experimental trials all entailed a 59 h fast. This duration of fasting was selected based on in our previous study [8] in which we found the robust fasting-induced elevations in the mean 24 h GH concentration and lipolytic rate were markedly suppressed with a high dose of GHRHa (10 mg/kg/h). These trials differed only by the contents of an infusion solution provided throughout the 59 h fasting period. Subjects were infused during the prolonged fasting period with either normal saline (“Control”; *n* = 15) or with graded doses of a GHRHa (Ac-Tyr1, d-Arg2; GHRH 1–29-amide; Bachem, Torrance, CA). The different GHRHa doses administered were: 10 μg/kg/h (“High”; *n* = 15), 1 μg /kg/h (“Medium”; *n* = 8), or 0.5 μg /kg/h (“Low”; *n* = 6). All subjects participated in the 12 h fast trial, the Control trial, and at least one dose of GHRHa trials. During the 12 h fast trial, subjects were provided with standard isocaloric diet (energy intake relative to body mass), divided into breakfast (0700 h), lunch (1200 h), dinner (1700 h), and a bedtime snack (2100 h). During all of the 59 h fasting trials, subjects were once again provided a standardized isocaloric diet on the day of admission, and then they fasted with only water allowed ad lib for 59 h. During all of these 59 h fasting trials, infusions of either saline (Control), or the different doses of GHRHa (Low, Medium, and High) were started after consumption of the dinner meal (1800 h) on the first day of admission. Blood samples were collected every 20 min during the final 24 h of the fasting period for measurements of plasma concentrations of GH, insulin and glucose. Assessment of the lipolytic rate was performed during the last 3 h of each 59 h fasting period using a primed steady-state infusion of [d5]-glycerol, as described in our previous publications [8, 10], and outlined briefly below. After completing the fast the subjects were fed and discharged from the hospital. The trials were conducted in a randomized order. Women were studied in the early follicular stage of their menstrual cycle (days 1–10 after the onset of menstrual bleeding), or during the “week off” of oral contraception pills (for habitual users). All women also underwent a urine pregnancy test before their participation in each experimental trial.

### Analytic methods

#### Stable isotope analysis

We infused trace amounts of [d_5_]-glycerol and measured lipolytic rate (rate of appearance (Ra) of [d_5_] glycerol in plasma) in the same manner as in our earlier studies [8, 10]. Blood samples were collected from heated arm (arterialized sample) [12] into pre-chilled tubes containing EDTA, centrifuged immediately and plasma was stored at –70C until analysis. The tracer:tracee ratio (TTR) of plasma glycerol was measured by electron impact ionization gas chromatography/mass spectrometry (GC/MS). GC/MS analysis for these substrates was performed as described by Patterson, et al. [11]. The TTR in plasma was used to calculate glycerol Ra using steady-state equations, as previously described [12].

#### Plasma hormone concentrations

Plasma GH concentrations were measured by chemiluminometric assay (Nichols, San Juan Capistrano, CA). Plasma insulin was measured by radioimmunoassay (RIA) (DSL, Webster, Texas). Plasma glucose was measured by glucose analyzer (Thermo Scientific, Middletown, Va., USA).

### Statistical design

GH profiles were analyzed by the CLUSTER program (SAS 2010 software) using t-statistics of 2 and a cluster size of 2 × 2 for pulse recognition. Based upon the sensitivity of our assay, minimal pulse/peak amplitude was set at 0.03 μg/L. Mean 24 h GH levels were calculated by averaging GH values from the subject’s 24 h GH profiles. Mean pulse amplitude was calculated from the maximal GH value within each pulse. Mean pulse mass/area was calculated as area under the curve between 2 flanking valleys in the profile. GH valley concentration was defined as GH concentration flanked by 2 CLUSTER-identified pulses. Mean nadir was calculated as the lowest 5% (4 measurements) of GH values from each profile.

Data groups were analyzed by ANOVA with Tukey’s post hoc analysis where appropriate. Correlation analysis was performed between discrete GH parameters and the rate of lipolysis. Data are shown as mean ± standard error (M ± SE). *P*-value of < 0.05 was considered statistically significant.

## Results

### Plasma glucose and insulin concentrations

Table 1 shows that compared with plasma glucose concentration after a 12 h fast (97 ± 2 mg/dl), more prolonged fasting lowered the mean plasma glucose concentration measured during the last 24 h of the 59 h fast by more than 20% (*P* < 0.00001 for all vs. 12 h fast). Plasma glucose concentration during the 59 h fast ranged between 70 and 80 mg/dl for all subjects, and there were no differences in plasma glucose concentration among the 59 h fasting trials. Accompanying the lower plasma glucose concentrations during the 59 h fast trials, mean plasma insulin concentration (measured during the last 24 h of the fast) declined from an average of 15.5 μU/ml to an extremely low at ~ 1–1.5 μU/ml, with no statistical difference among any of the 59 h fasting trials.

**Table 1:** 24 h mean plasma insulin and glucose concentrations

		59 h Fast			
	12 h Fast (No GHRHa) [*n* = 15]	Control (No GHRHa) [*n* = 15]	Low (0.5 μg/kg/h GHRHa) [*n* = 6]	Medium (1 μg/kg/h GHRHa) [*n* = 8]	High (10 μg/kg/h GHRHa) [*n* = 15]
**Insulin** (μU/ml)	15.5 ± 3.5	1.5 ± 0.1*	1.4 ± 0.3*	1.6 ± 0.2*	1.4 ± 0.2*
**Glucose** (mg/dL)	97 ± 2	77 ± 2*	75 ± 3*	76 ± 1*	75 ± 2*

*Significantly lower than 12 h Fast, *P* < 0.05. GHRHa: “Growth Hormone Releasing Hormone-Antagonist”

### Plasma growth hormone and lipolysis values

**Fig. 1:**
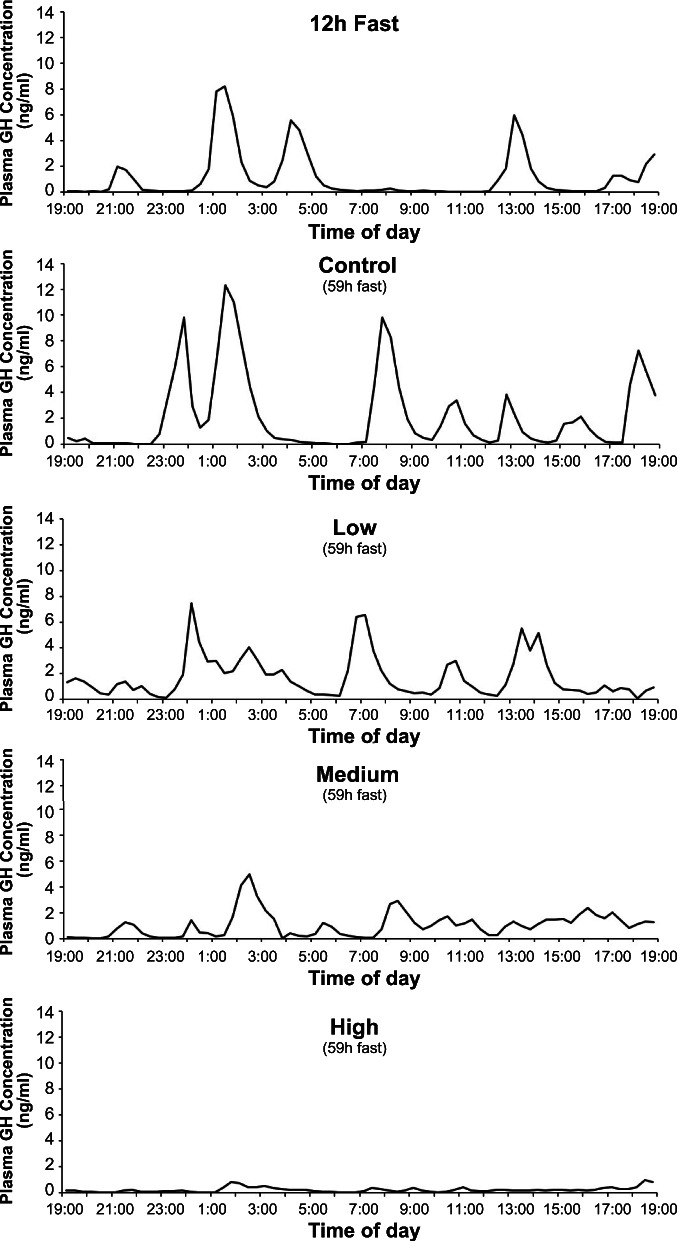
Plasma GH profiles in a subject who underwent all 5 stages of the protocol. Note increase in GH peak frequency and amplitude during 59 h fast, and the dose-dependent decline in GH pulse amplitudes with increasing doses of GHRHa

Figure 1 presents actual GH values obtained in a subject who went through all stages of the entire protocol. Note the increases in GH peak frequency and amplitude after 59 h fasting.

**Fig. 2:**
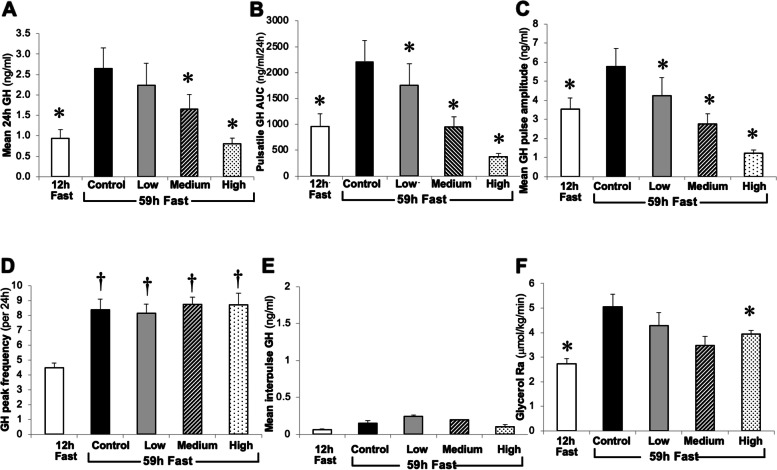
Composite data of discrete parameters of GH pulsatility and Glycerol Ra (rate of lipolysis) for all stages of the protocol. *p < 0.05 vs. Control (59 h fast without co-administration of GHRHa). †*p* < 0.05 vs. 12 h Fast. Lipolytic rate was higher after 59 h fast/Control vs 12 h fast (*P* = 0.001). GHRHa infusions at low and medium doses during 59 h fast were associated with numerically but not statistically lower lipolytic rate compared with 59 h fast with no GHRHa (Control). High-dose GHRHa infusion during 59 h fast significantly lowered lipolytic rate compared with 59 h fast/Control

Figure 2 shows mean 24 h GH concentration and parameters of GH pulsatility (i.e., pulsatile GH AUC, mean GH pulse amplitude, GH peak frequency, and mean interpulse GH concentration) after a 12 h fast and after a 59 h fast without GHRHa (Control) and after 59 h fasts with Low, Medium, and High GHRHa infusions. As expected, the 59 h fast markedly increased endogenous plasma GH concentration (Control vs. 12 h fast; *P* = 0.0044). GH peak frequency during fasting increased from 4.5 ± 0.3 to 8.4 ± 0.7 pulses/24 h (Fig. 2D; *p* < 0.001) and remained stable during GHRHa infusions at all doses thereafter (8.2 ± 0.6; 8.8 ± 0.5; 8.5 ± 0.6 pulses/24 h, *p* > 0.05). Mean GH pulse amplitude went up during 59 h fast from 3.5 ± 0.6 to 5.8 ± 0.9 ng/ml (Fig. 2C; *p* < 0.001) and declined in a dose dependent manner during graded GHRHa infusions (p < 0.001) and pulsatile GH AUC increased during fasting (Fig. 2B; *p* < 0.001) and declined in a dose-dependent manner during administration of GHRHa (p < 0.001) despite stable GH peak frequency. The mean interpulse GH concentration did not change throughout the protocol (Fig. 2E; *p* > 0.05). Therefore, changes in total GH production were due to changes in the GH pulse amplitude and not to the GH peak frequency or baseline GH concentrations.

Lipolytic rate increased nearly 80% between the 12 h fast and 59 h fast (Control), and GHRHa administration blunted this fasting-induced increase in lipolytic rate in a dose-dependent manner (Fig. 2F). The highest dose of GHRHa infusion (10 μg/kg/h) significantly reduced lipolytic rate vs. Control (Fig. 2F), whereas the decline in lipolytic rate during Low and Medium GHRHa infusions did not reach statistical significance. Intersubject variability and relatively low number of subjects in the “Low” and “Medium” groups likely were responsible for the reduction in lipolytic rate to not reach statistical significance.

### Relationships between lipolytic rate and discrete parameters of GH pulsatility

**Fig. 3:**
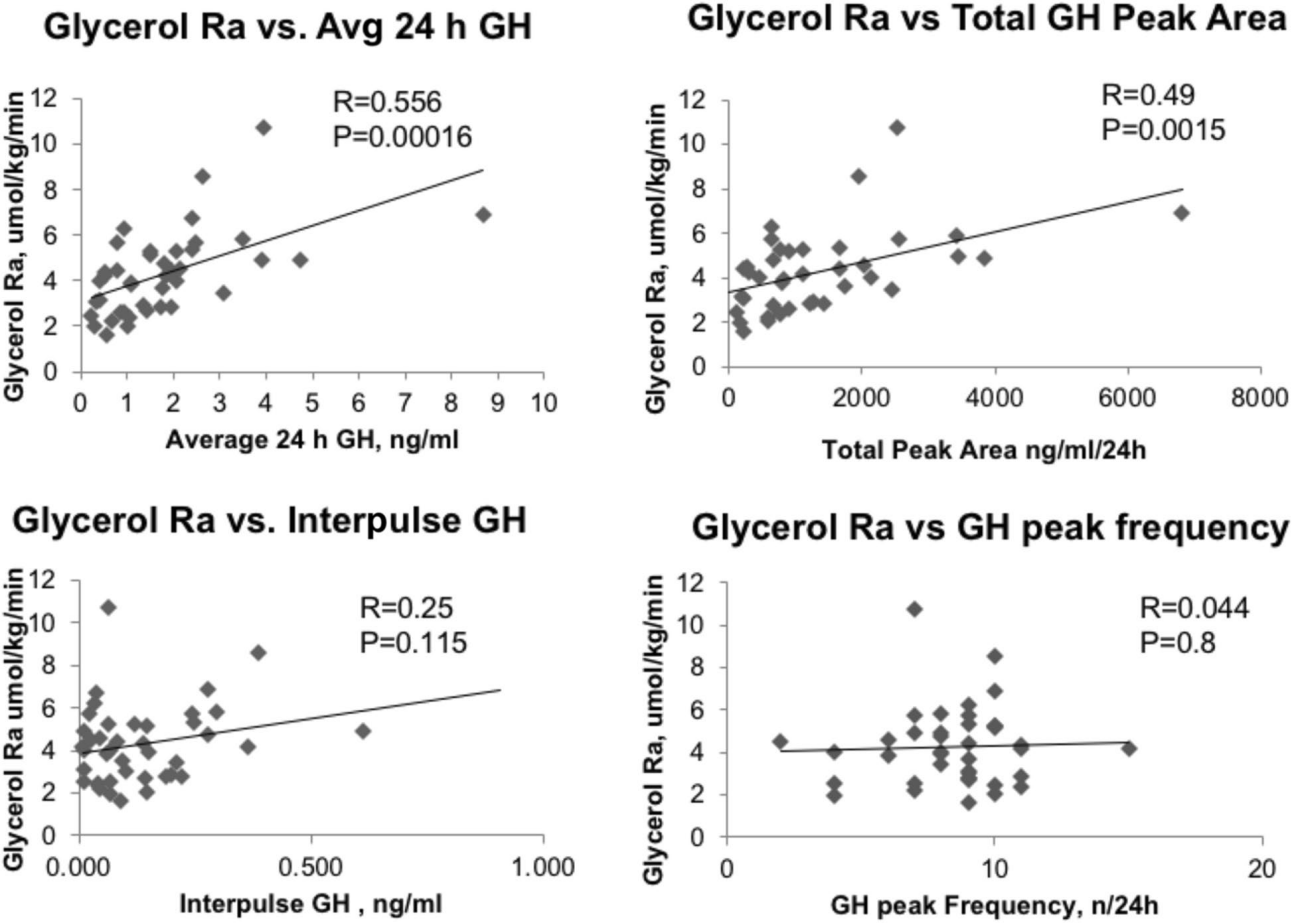
Correlation analyses of mean 24 h GH concentration, pulsatile GH AUC, GH peak frequency and inter-pulse GH concentrations vs. rate of lipolysis. Correlation coefficients and levels of significance are shown in the Figure panels

To help ascertain how discrete components of GH secretion pattern may influence lipolytic rate during fasting, we performed correlation analyses (Fig. 3) between the individual parameters of GH pulsatility and lipolytic rate using the data from the 59 h fasting groups only (Control and all doses of GHRHa). There was a strong correlation between the lipolytic rate and mean 24 h GH concentration (R = 0.556, *p* = 0.00016), as well as total GH peak area (i.e., pulsatile GH AUC) (R = 0.49; *P* = 0.0019), but not with mean interpulse GH concentration (R = 0.25; *P* = 0.115) or GH peak frequency (R = 0.044; *P* = 0.8).

## Discussion

This study shows that the rate of lipolysis correlates tightly with only pulsatile GH output (i.e., pulsatile GH AUC) and not with interpulse GH concentrations or GH peak frequency. Traditionally it is thought that increased lipolysis and ketosis of starvation are the result of cessation of insulin production with fasting [13–16]. However, we have previously reported that inhibition of mean daily GH by about 30% using a relatively high dose of GHRHa (10 μg/kg/h) during prolonged fasting significantly attenuated the fasting-related increase of the lipolytic rate, despite virtually complete suppression of insulin secretion [8]. Therefore, the observed changes in rate of lipolysis during prolonged fasting were the direct result of the fasting-induced elevation in GH secretion. In support of our data, Moller et al. [17] found that blocking GH receptors with pegvisomant selectively suppressed lipid mobilization and oxidation after 36 h fast. However, since pegvisomant induces global suppression of GH action, that study could not address the influence of the discrete components of GH pulsatility.

Over the past several years, it has become evident that the pattern of GH presentation to the peripheral tissues plays an independent and tissue-specific role in mediating the resultant metabolic effects [18]. For example, pulsatile patterns of GH secretion are important for controlling hepatic expression of P450 enzymes in both humans and rats [18, 19]. In addition, pulsatile GH administration to male rats increased cartilage and muscle IGF-1 mRNA levels, but steady state GH administration did not [20]. More relevant to our present findings, Cersosimo et al. [21] demonstrated that pulsatile, but not continuous, GH administration to humans increased the rate of lipolysis, but this study did not address the issue of potential roles of GH peak frequency or of the baseline component of GH secretion. Similarly, we previously reported that only pulsatile GH administration in humans increased the rate of lipolysis [10], while plasma levels of IGF-1, reflecting its hepatic production, as well as muscle IGF-1 mRNA levels are controlled primarily by the interpulse, nadir, GH concentrations [9, 10].

In the present study, graded GHRHa doses induced a reduction in the fasting mean 24 h GH concentrations by 10–88%. We found strong correlation between rate of lipolysis and mean plasma GH concentration during the 24 h period immediately preceding our measurement of lipolytic rate. This was in accord with our earlier findings [8]. However, discrete analysis of GH pulsatile parameters revealed that mean GH pulse amplitude and total GH total peak area may largely underlie this effect. We found a strong correlation between the rate of lipolysis and mean 24 h GH concentration (R = 0.556; *P* = 0.00016), and total GH peak area (R = 0.49; *P* = 0.0015), but not the interpulse GH concentrations (R = 0.25; *P* = 0.115) or GH peak frequency (R = 0.044; *P* = 0.8). Thus, neither GH peak frequency nor the interpulse GH levels had any association with the reduction of the rate of lipolysis during the fasting state. In this study we used as our main parameter pulsatile GH AUC rather than GH pulse amplitude. The former gives us more accurate estimate of GH secreted during the pulse by integrating all GH values found during the secretory episode, the duration of the pulse and the total number of secretory pulses rather than a single GH value of pulse amplitude. In addition, GH pulse AUC removes the interfering factor of the underlying baseline, tonic GH background. Therefore, this study confirms our earlier findings that the pulsatile (as opposed to the tonic) component of GH secretion is the specific regulator of fat metabolism during prolonged starvation. Moreover, the present study expands on these findings by demonstrating dose-dependence of the effect of suppressing pulsatile GH output during fasting and the decline in the lipolytic rate.

We have previously shown that in humans it is the nadir GH concentrations that determine the magnitude of plasma IGF-1 concentrations (mainly of the hepatic origin) as well as muscle IGF-1 mRNA abundance [9, 10]_._ The current study was not designed to revisit this question since the model of prolonged fasting is not appropriate to address that issue: fasting per se decreases plasma IGF-1 levels [22] and would be a major confounding factor in analyzing a model of central GH inhibition.

The mechanism(s) regulating the increased GH pulsatile component during fasting are still unclear. Fasting-induced decrease in circulating IGF-1 concentrations [22] is likely to be involved, since the negative feedback of IGF-1 on GH secretion specifically suppresses GH pulse amplitude [22–24]. Also, the reduction in insulin secretion with fasting may also play an important role, because elevated insulin concentration during overeating rapidly and specifically suppressed GH pulse amplitude [25]. These two mechanisms may work independently of each other, since the decline of circulating GH by IGF-1 infusion occurred despite rapid insulin suppression [22]. Therefore, both insulin and IGF-1 receptors may participate separately and, potentially, by different regulatory pathways.

In summary, our major findings indicate that the pulsatile component of the elevated GH secretion during fasting is a specific regulator of the increase in rate of lipolysis during the prolonged fast. As we have shown previously, GH assumes its role as a primary lipolytic stimulator only in the presence of markedly inhibited insulin concentrations [8]: an ~ 30% inhibition of GH output in normally-fed individuals had no effect on the rate of lipolysis. Importantly, fasting-induced stimulation of GH pulsatility may be a compensatory mechanism aimed at mobilizing endogenous energy to help assure survival. In contrast, restoration of pulsatile GH profile during energy overabundance appears to be deleterious, leading to insulin resistance and hyperlipidemia [26] . Therefore, understanding the physiological mechanisms of GH regulation and action during different nutritional situations is important for the interpretation of parameters of hormonal milieu and constructing sound therapeutic strategies in clinical situations associated with under-, or overnutrition.

## Abbreviations

GHRHa: GHRH receptor antagonist
GH: Growth hormone
AUC: Area under the curve
